# Attitudes toward Wind Power in Norway–Solution or Problem in Renewable Energy Development?

**DOI:** 10.1007/s00267-023-01870-5

**Published:** 2023-08-31

**Authors:** Bjørn P. Kaltenborn, Rose Keller, Olve Krange

**Affiliations:** 1 Norwegian Institute for Nature Research, Vormstuguvegen 40, 2624 Lillehammer, Norway; 2 Norwegian Institute for Nature Research, Sognsveien 58, 0855 Oslo, Norway

**Keywords:** Wind power development, Environmental attitudes, Energy policy, Ecomodernism, Degrowth, Landscape values

## Abstract

Wind power has become an increasingly important source of renewable energy in Norway. Current demand and production capacity have exceeded expectations stipulated in energy policies a few years back. Wind power affects landscape characteristics, and the rapid development has created considerable public conflict. However, knowledge to date about public attitudes toward wind power development in Norway is limited. We surveyed a representative sample of the Norwegian public to examine relationships between wind power development and place attachment, localization, and policies. We also examined if attitudes toward wind power are linked to broader environmental attitudes and meaning of place. Public attitudes range from strong support to strong opposition. We found limited support for NIMBY effects. Support versus opposition correlates with attitudes toward place attachment, localization of wind power plants and energy policies. We found evidence of a dichotomy between the more fundamental world views of eco-modernism versus de-growth influencing the more specific wind power attitudes. We argue that policy institutions have underestimated the power of attitude diversity in the wind power debate, and that social acceptability of future wind power development will depend on improved understanding of how social values of landscapes are impacted.

## Introduction

Wind power development has emerged as a major landscape transforming agent in Norway in a relatively short time. How does this affect public opinion, and can wind power become the promised part of the solution to the problem of global warming? As recent as a decade ago, wind power was not considered a major, future energy supplier, but rather a limited supplement to hydropower. Now the discourse is changing.

As modern societies continue to increase demand for energy, wind represents a renewable form of energy that legitimizes projects aiming to mitigate climate change through the green growth discourse. However, while windmills can drastically change the character of the landscape, they may also be seen as progress on behalf of the environment. Technological solutions of this kind fits well within the optimistic and growth-friendly paradigm of ecological modernization. In the perspective of eco-modernization climate change mitigation is fundamentally a technological challenge, and an achievable goal that in no way conflicts the dominant economic growth regime. From an ‘ecomodernist’ perspective, ‘green growth’ will result in necessary emissions reduction through moderate system change and technological innovation (Hayden 2014, Asafu-Adaje et al. 2015). Obviously, to consider that environmental issues can be managed within the existing social and economic structure is a contested political standpoint and strategy, albeit not by the political elite. The Norwegian national political discourse seems to be enclosed in a dominant cross-party consensus that environmental degradation can be resolved within a system that demands endless economic growth.

An opposing position is found within the degrowth movement (Healey et al. 2015, Hickel and Kallis 2020). As opposed to the ecological modernization and green growth idea, the degrowth movement recognizes that fundamental social and economic change is required, accusing ecological modernization to offer nothing else than to “sustain the unsustainable” (Fournier 2008) From a degrowth perspective, cutting back on carbon emissions is in essence an issue that requires fundamental social and cultural shifts to lower levels of consumption. Hence, degrowth represents more traditional environmental attitudes where growth and environmental protection are seen as opposites. Little is known about how these fundamentally opposite positions impact popular assessments of wind power developments.

This study targets a major knowledge gap in the understanding of how the public relates to the rapid increase in wind power development. The contribution of the study runs along two lines. We present a nationally representative picture of the public’s current perceptions of different aspects of wind power development in Norway, and secondly, we examine the relationships between these wind power-specific attitudes and the public’s general environmental attitudes to better understand what drives public judgment as a source of renewable energy for the future.

We ask two questions: 1) What are the attitudes of the Norwegian public towards the links between wind power development and place attachment, policies, and localization of wind parks? and 2) To what extent are the public’s attitudes toward wind power development a discrete attitude complex, independent of general environmental attitudes, or conversely embedded in broader sets of attitudes and meaning attributed to place? Moreover, we seek to unravel the association between wind power attitudes and what might be seen as an antagonistic position within the broad environmental movement, corresponding to the conflicting positions between ecological modernization and degrowth. We hypothesize that attitudes towards wind power developments varies with fundamental views on environmental measures and policies.

### Wind Power Development in Norway

The public energy policy of the early 2000’s set a target of 3 terawatt hours (Twh) by 2020. The answer in 2021 is 11,8 Twh (NVE 2022). The unexpected rise in wind power development comes as result of rapid changes in technology, demand, markets, global energy supply and shifting energy policies, including a stronger integration in international energy agreements in attempts to address the demands of the green transition and climate change mitigation. Currently 70 wind power plants with 1354 turbines are spread out along large sections of the Norwegian coastland and some inland locations with visual, audial, and cumulative environmental impacts far beyond the construction zones (NVE 2022).

The development of wind power in Norway has progressed through three more or less distinct phases. During the initial phase ca. 1998–2008, the focus was on developing technology and establishing a regulatory system for a new source of energy. Between 2008 and 2018, emerging Norwegian policy was increasingly linked to EU’s renewable energy policies and international agreements on climate change mitigation. Norwegian policies were aimed at reducing conflict, increase profitability and predictability for new markets and secure Norwegian investments. The third phase is ongoing as wind power construction is currently encountering major hurdles for continued expansion (Vasstrøm & Lysgård 2021). This may be changing now, however. As the war in Ukraine drive energy prizes to the roof, opposition to wind energy developments may decline.

Even though wind power constitutes a mere 7,5 per cent of Norway’s energy production currently (NVE 2022), wind power plants have industrialized landscapes across large parts of Norway evoking widespread public reaction to the extent that the government in 2019 chose to withdraw a national level master plan for further wind power development and all current applications for new concessions are put on hold (NVE 2022).

Various interest groups have become increasingly vocal. A decade ago, formal public policy stated that renewable energy development in Norway should be organized so that the supply situation remains secure, natural resources shall be utilized for value creation, environmental concerns must be considered, and the policies should stimulate efficient use of energy (NOU 2012). A few years later, policies put more emphasis on climate change and cooperation and integration with the European energy market and the need for increase in wind power production (MoPE, 2015). At this stage, local conflicts had started to escalate and a new energy policy from 2016 also highlighted the need to reduce conflicts and increase the predictability for both local communities and actors in the market (Ministry of oil and energy 2016). Several interest groups and organizations called for a better knowledge base and overall plans for development which would facilitate assessments of cumulative impacts. The World Wildlife Fund for instance, recently commissioned a report which showed that the vast majority of existing wind plants violate what they considered essential criteria for allowing concessions (Nowell et al. 2020).

Estimating the influence of wind power plants on the landscape is a tall order (Zerrahn 2017). The question may seem simple but contains a range of specific queries into questions like direct area coverage, structural effects like fragmentation, connectivity, threat to wild mammals and birds, ecological integrity, spatial configuration of non-impacted parcels of land, noise, disturbance, visual impacts, species specific impacts and cumulative impacts. Hence, wind power developments in Norway often conflict human practices and interests, such as hunting, outdoor recreation, reindeer husbandry and environmental interests. As of today, the development is spatially extensive in the sense that wind power parks are spread out along large sections of the coastline and with some development in inland regions. In terms of direct seizure of land, the current windmill parks cover approximately 500 km2 as laid out in formal plans and concessions. This is estimated to reach approximately 800 km2 when existing plans are completed by 2022 (NVE 2022), or possibly later due to political inertia in solving existing conflicts. However, this estimate of spheres of influences takes no account of effects beyond direct acreage. There is a great lacuna of knowledge about a range of socio-ecological impacts and especially long-term and cumulative impacts.

In this paper we are motived by two different, but related aspects of this type of renewable energy development. First, since windmills have made their mark on large sections of the Norwegian coastline and some inland mountain areas, we assume that wind power installments change the experience of their surroundings and people’s perception of place. We consider place as more than physical space or individual cognitive experience, and lean more towards place as social representations embedding symbolic meaning and knowledge that develops as individuals and institutions communicate over time (e.g., Devine-Wright and Howes 2010). In this perspective, local opposition to wind power has been conceptualized as a type of place-protective action when technological interventions in the landscape impact pre-existing emotional attachments and place-identity processes (Devine-Wright 2009).

The second premise is that public attitudes toward new phenomena in the environment (and otherwise) are highly malleable until the public has had time to digest the changes and come to more coherent judgement, and this takes time (Heberlein 2012, Bidwell 2013, Bisconti 2018). Attitudes are unstable until a critical mass of people has direct experience, or enough of the public has been convinced that their core values are connected to for instance wind power as a landscape-changing agent. Object-specific attitudes can also have relations to broader environmental attitudes, i.e., psychological constructs of how we view our complex surroundings (Milfont and Duckitt 2010). In this paper we explore the assumption that the public’s relatively newly formed and malleable attitudes toward wind power can be correlated with different aspects of environmental world views.

### Research on Wind Power

Wind power development is perhaps the foremost example in recent times of how renewable energy tends to create conflicts between national interests and local communities (Hagget 2011, Oles & Hammarlund 2011). Scientific and anecdotal/popular evidence shows that over the last twenty years, i. e. since the inception of the ‘wind power era’ in Norway, public attitudes toward this development have been dynamic. Several studies have addressed different aspects of the wind power situation in Norway such as the role of political orientation (Karlstrøm and Ryghaug 2014), policy (Buen 2006, Vasstrøm and Lysgård 2021), community acceptance (Dotterud Leiren et al. 2020), community perspectives and local participation (Thygesen and Agarwal 2014, Inderberg et al. 2019, Staupe-Delgado and Coombes 2020), planning processes and regulatory mechanisms (Petterson et al. 2010, Blindheim 2013, 2015), offshore development (Gulberg 2013, Steen and Hopsdal Hansen 2018) and impacts on cultural heritage (Jerpåsen and Larsen 2011). However, only a few studies have investigated specific attitudes toward wind power development in Norway (Solli 2010, Rygg 2012). Systematic research on attitude trends is lacking, and there is a need for studies into attitude development over time as well as more representative reports on public perceptions.

Social acceptance of wind power development is an expression of an attitude complex that comprises both the experience of a changing landscape and underlying, more basic beliefs about normative aspects of the environment (Meyerhoff et al. 2010, Johnsen Rygg 2012, Wolsink 2018, Early research on attitudes towards wind power often assumed that opposition could be attributed to the NIMBY – not in my backyard effect (e.g., Bosley and Bosley 1988). However, there is relatively strong consensus among researchers that the NIMBY concept is too simplistic and incapable of explaining the underlying motivations, beliefs and concerns that can lead to negative as well as positive attitudes toward wind power (e.g., Devine-Wright 2009, Rygg 2012, Rand and Hoen 2017, Vasstrøm and Lysgård 2021). In fact, many social science researchers now seem to agree that the concept of NIMBY should be shelved as an explanation for opposition to wind power development (e.g., Wolsink 2006, Petrova 2016). A study based on data from multiple European countries documents the complexity of community acceptance of wind power and how it is linked to technical characteristics, social, economic, and environmental impacts, individual characteristics (Hadler et al. 2022), and not the least the specific nature of community acceptance (Dotterud Leiren et al. 2020). A review of thirty years of wind power related research in North America also emphasized the complexity and interrelatedness of socioeconomic and environmental factors, sound and visual disturbance, the specific context, as well as issues of fairness, participation, and trust in the development processes. This review also showed that North American support for wind power has remained high during the last thirty years (Rand and Hoen 2017), but varies by region (landscape factors), perceptions and trust in management practices (Ferguson et al. 2021).

Earlier research has produced considerable new knowledge about the diversity in environmental attitudes (Franzen and Vogl 2013, McIntyre and Milfont 2016, Somerwill and Wehn 2022) and the heterogeneity of the environmental movement (e.g Wolsink 2010, Healy et al. 2015). We assume that this will also be reflected in attitudes toward wind power. For instance, we would expect that differences between eco-modernists and techno-optimists on the one hand and de-growth proponents are reflected in a specific issue like wind power since they have widely different notions of solutions to the energy and climate challenges (e.g., Kerschner et al. 2015, Kish and Quilley 2017, De Beukelaer 2022).

## Methods and Data Collection

We collected data through an online questionnaire during June–July 2021 using an established online survey platform and panel (Qualtrics, qualtrics.com). The sample was designed to be nationally representative of the Norwegian public and stratified in terms of age, gender, and region. We collected 1220 fully completed responses; a response rate of 83 per cent. Of the 1220 survey respondents, 48% identified as female and 51% male The mean age was 46, and mean years of education was 15 (42% of the sample had over 15 years). The majority reported their annual household income between 400–600 k NOK with a mean of 800 k, which is near the mean two-earner household income reported nationally (890 k, Statistics Norway 2022).

Because environmental attitudes are closely related to social status (Milfont and Duckitt 2010), we asked respondents to provide their childhood socio-economic status (SES) based on an item set of signaling social status through book collections, musical instruments, and leisure. The sample was evenly split between low and mid- SES, (31 and 29% respectively), and 40% reported high SES, meaning respondents grew up with all three aspects of social status (books, instruments, leisure). The political ideology of the sample followed the relative representation of parties in the National Parliament: 31% of the sample adheres to a conservative party (11% to the extreme right). Our sample reported relatively even and ‘neutral’ levels of trust in ‘everyday folk’ (M = 5.6; scale: 1–10), public authorities (M = 5.4) trust in local authorities (M = 4.8) and the lowest trust in energy business and developers (M = 4.5). In terms of participation in environmental issues associated with wind energy, over a third of the respondents spent time learning about wind energy issues in the past five years (38%), though fewer reported active participation in environmental organizations (14%) or participating in a wind energy protest (11%).

We measured attitudes toward wind power development in different ways. To tap into the overall view of wind power we first asked whether the respondents liked or disliked the construction of wind power in Norway in general. This was followed by the question “What do you think about the construction of wind power close to where you live?” Answers to both questions were given on a 5-point Liker scale ranging from 1 (like very much) to 5 (dislike strongly). Then followed a set of twelve specific statements about wind power development where the respondents were asked to indicate to what extent they agreed or disagreed with each of them. Again, we used a Likert scale. This time ranging from 1 (disagree completely) to 5 (agree completely). This segment of the questionnaire covered three different aspects of wind power development: place attachment, relations to politics, and localization of wind power plants (For a detailed overview of the actual statements see Table 2). The statements were based on review of the scientific literature and popular reports (newspapers, Internet) from recent conflicts associated with the construction of wind power plants in Norway.

We measured different aspects of broader environmental attitudes employing a newly developed instrument aimed at capturing the complexity of how people view the environment on the level of environmental worldviews (Kaltenborn et al. 2021). The attitudes toward wind power development comprise measurements of what people think about a specific landscape intervention, while the measurement of environmental attitudes tap into more stable attitudinal constructs. The scale was developed as a response to the rapidly changing concepts of ‘environment’ in contemporary society. Most of the previously existing scales such as the New Environmental Paradigm Scale (Dunlap and Van Liere 1978, Dunlap 2008), the ‘Ecology scale’ Maloney and Ward 1973, and the ‘Environmental Concern scale’ (Maloney et al. 1975), are today incomplete and/or even outdated in terms of how the political discourse on environment has developed. Many earlier scales are too coarse-grained to capture the diversity in attitudes we see today across the political spectrum, such as for instance the schism between eco-modernism and degrowth movements (e.g., Wolsink 2010). The newly developed scale that we use here recognizes that it is difficult, and probably not valid to measure environmental world views as a single dimension or higher-order attitude.

The original scale comprises seven main dimensions; Responsibility, Public relations, Nature’s values, Wild animal rights, Economy, Technology and Use and protection (Kaltenborn et al. 2021). In this study we used four of these dimensions: Nature’s value, Economy, Technology, and Use and protection with three items for each dimension. The respondents were asked to rate their level of agreement to each of the twelve statements. Conceptually, these dimensions and underlying items (three for each dimension) correspond to the ‘eco modernization’ (EM) versus ‘traditional environmental attitudes’ (TEA) divide. The concept of eco modernization argues that nature is robust, that economic growth trumps, but is also compatible with conservation, and that technology is the answer to environmental challenges. The specific statements we used as proxies for EM were: “Economic growth is more important than environmental concerns”, “It is more important to have the opportunity to use natural resources to produce goods and services than protect nature”, “It is more important to create economic growth than to protect nature”, “Most environmental problems can be solved by using new and better technology”, “Technology will ensure a sustainable society in the future”, “Technological development solves more problems than it creates”, “Future environmental solutions will be created through economic growth”, There is no contradiction between a climate-friendly future and the consumption level we have today”, and “Economic growth is a prerequisite for a successful green transition”. Conversely, the traditional environmental attitude orientation posits that nature has intrinsic value, that economic growth and environmental protection is a contradiction, and that human impact to nature leads to emotional disturbance and loss of meaning. The statements we used as proxies for TEA included: “Nature has value in and of itself”, “The intrinsic value of nature is more important than the extracted resources for industry”, and “It makes me sad to see large-scale development in nature”.

We used SPSS (version 27) for data analysis. General attitude patterns were examined descriptively (Table 1). Relationships between general attitudes toward wind power as the independent variable and specific attitudes toward wind power was examined using ONEWAY analysis of variance. We grouped the general attitude variable into three categories (Like very much and Like=Like, Neutral, and Dislike and Dislike very much=Dislike) (Table 2). We assumed that general and more stable environmental attitudes influence specific attitudes toward wind power more than the other way around, and hence calculated an index for each of the four main dimensions and used these as independent variables in ONEWAY analysis of variance with the specific wind power attitudes as dependent variables (Table 3).

**Table 1:** General attitudes toward wind power in Norway (in per cent)

	What do you think about the construction of wind power in Norway in general		What do you think about the construction of wind power close to where you live	
	%	*N*	%	*N*
Like very much	14,7	179	10,4	126
Like	21,6	263	17,4	212
Neutral	29,2	355	28,0	340
Dislike	17,0	207	20,2	246
Dislike strongly	17,4	212	24,0	292

**Table 2:** Attitudes toward specific aspects of wind power development and differences across groups liking (L), neutral (N) and disliking (D) wind power development in general (ONEWAY Analysis of variance, mean scores)

Statements	Wind power attitude segments	Mean scores	F-values	Sign.	*N*
*Place attachment*					
Windmills are a foreign element in Norwegian nature	L	3,46	225,212	0000	442
	N	3,77			355
	D	5,22			418
I am sad when I see the construction of windmills in previously untrammeled natural areas	L	2,98	410,718	0000	442
	N	3,07			355
	D	5,35			418
Windmills degrade my experience of being attached to nature	L	2,93	312,906	0000	442
	N	3,51			355
	D	5,09			418
Windmills change the meaning I find in landscapes	L	3,08	272,871	0000	442
	N	3,52			355
	D	5,06			418
*Relations to politics*					
Development of wind power will allow us to meet future energy needs without reducing energy consumption	L	4,27	305,896	0000	442
	N	3,60			355
	D	2,32			418
Wind power can replace oil and gas with time	L	4,00	178,486	0000	442
	N	3,43			355
	D	2,39			418
We should develop more wind power so that we electrify oil- and gas extraction on the continental shelf	L	4,16	301,398	0000	442
	N	3,52			355
	D	2,17			418
It is good that Norway participates in an international energy market	L	4,44	120,558	0000	442
	N	3,96			355
	D	3,00			418
*Localisation*					
We should build more wind mills on the Norwegian continental shelf	L	4,52	182,729	0000	442
	N	3,68			355
	D	2,75			418
Wind mills should be located in areas already impacted by roads and houses	L	3,88	105,897	0000	442
	N	3,34			355
	D	2,52			418
Some parts of the country should be spared from wind power parks	L	4,00	132,476	0000	442
	N	4,27			355
	D	5,35			418
Development of wind power is fine as long as it doesn’t cover unacceptably large areas	L	4,23	227,762	0000	442
	N	3,97			355
	D	2,53			418

Response format: 1: Completely disagree, 2: Disagree, 3: Neither agree nor disagree, 4: Agree, 5: Completely agree

**Table 3:** Relationships between attitude segments toward wind power development (Like, Neutral, Dislike) and general environmental attitudes (mean scores)

Environmental attitude dimensions	Wind power attitude segments	Mean scores	F-values	Sign.	*N*
Nature’s value	L	4,59	37,874	0000	442
	N	4,70			355
	D	5,11			418
Economy	L	3,24	27,790	0000	442
	N	2,86			355
	D	2,66			418
Technology	L	4,07	9597	0000	442
	N	3,96			355
	D	3,78			418
Use and protection	L	3,80	17,243	0000	442
	N	3,58			355
	D	3,38			418

Response format: 1: Completely disagree, 2: Disagree, 3: Neither agree nor disagree, 4: Agree, 5: Completely agree

## Results

### General attitudes toward wind power

Table 1 shows the distribution of the overall view of the public towards wind power in Norway. When asked about wind power development in general, without specifying locations or distance to people’s homes, we find a near normal distribution between those who support it, are against it or remain neutral in this question. 36.3 per cent claim they like or like very much that wind power construction goes on in Norway. Slightly less than one-third of the sample have a more indifferent or neutral view, while 34,4 percent dislike or strongly dislike the development of wind power. When we focus the same question on respondents’ opinion of wind power development close to where they live, the pattern changes somewhat, with approximately 28 percent supporting or being neutral and around 44 per cent opposing this kind of development (Table 1). This shows that the Norwegian public are evenly distributed across the attitude spectrum when it comes to the general perception of wind power, without being particularly skewed towards support or opposition. Hence, when we qualify the question by linking it to proximity of residence, meaning people will have more direct experience with the windmills, the pattern becomes more skewed towards a negative attitude toward wind power development. However, the shift in attitudes is conceptually quite small, and in our opinion not sufficiently large to suggest a clear NIMBY effect.

### Relationships between attitudes toward wind power and place attachment, politics, and localization

The respondents were asked to rate their level of agreement with four statements about the effects of windmills on different aspects of place and landscapes (Table 2). Here we used the index computed from the general attitude towards wind power with three roughly equal size groups: Like, Neutral or Dislike. All the statements had a negative wording, i.e. wind power development was poised as having a negative, degrading or alienating effect.

There is a consistent pattern in how attitudes toward wind power development are linked to effects on place and landscape. For all the statements we find statistically significant differences among the three segments in the sample. Those who in general dislike wind power development are significantly more in agreement with the potentially negative effects of wind power development on the landscape than the two other segments. Conversely, those more in support of wind power development have less negative views of these effects. The neutral group places in between the supporters and opposers on all the statements (Table 2).

When we ask how general wind power attitudes relate to different aspects of policy and political decisions, we also find a consistent pattern. Here we also used four statements which all, in different ways, support the role and potential of wind power in Norway’s energy needs. Those who are generally in favor of wind power are in significantly higher agreement with these statements than the neutral and opposing segments. The differences are highly statistically and conceptually significant for all four statements (Table 2).

Much of the controversy over wind power development has centered on the localization of windmills. We presented the respondents with another four statements, covering aspects of localization such as proximity to areas already impacted by development and infrastructure and development on land versus the ocean. Again, we find an attitude pattern that resembles that of place attributes and political elements. Those who generally support wind power development see less problems with locating windmills close to existing roads and settlements, they are less concerned about area impacts and coverage, but agree less than the opposers and the neutral group that some parts of the country should be spared from wind mill parks. For all four statements about localization, we also find highly significant statistical differences (Table 2).

### Relationships between wind power attitudes and general environmental attitudes

The next step in the analysis was an examination of how broader environmental concern is associated with the public’s attitudes toward wind power (Table 3). Environmental concern or general environmental attitudes is a complex concept which here is broken down into four main dimensions. *Natures’s value* addresses the intrinsic values of nature, and the role of environmental conditions as an expression of the society’s character. *Economy* prioritizes economic growth and value creation from nature and natural resources over environmental protection. *Technology* covers techno-optimism and improved technology as the key to a sustainable future. *Use and protection* covers continued economic growth and consumption as prerequisites for the green transition and can be labelled as a form of eco-modernism.

We find significant differences in the respondents’ relationship between wind power attitudes and all four environmental attitude dimensions (Table 3). For the Nature’s value dimension, there is a clear gradient in the importance of this dimension with the opposers expressing this more strongly than the neutral and supporter groups. The reverse pattern goes for the Economy, Technology and Use and protection dimensions, with stronger emphasis on the importance of economic growth from the wind power supporter group than the neutral and opposer groups (Table 3).

## Discussion

The data collection and analysis in this study was conducted prior to the Russian invasion of Ukraine in February 2022. The war is currently creating turbulence end increasing prices in international energy markets. The ‘energy supply’ discourse in Europe is currently on the forefront of the political agenda. We anticipate that the precarious energy situation will influence attitudes toward renewable energy supply, emergency preparedness and contingencies among the public. We will need time to sort out what may be fleeting responses to perceived emergencies, high energy costs for much of the public, and what constitutes more lasting attitudinal changes. An obvious question to ask when more stable power balances are eventually achieved in the energy market is; will the public(s) become more accepting of landscape changes due to wind power expansion in exchange for stability in trading partners and energy costs? Suffice to say at this point that the relative weight of different attitudinal position in the Norwegian public may shift. However, we have no reason to believe that the general pattern of attitudes with diversity in terms of support and opposition, and the clear divide between eco-modernization/techno-optimism vs. degrowth/conservation, has changed significantly. Indeed, there has been little talk of consuming less energy in the long term, only conservation in the short term, suggesting stability in general attitudes towards the environment. In other words, we portend that the basic nature of relationships that we have identified here remain valid.

In this study we identified both broad patterns in the public’s attitudes toward wind power construction in Norway, as well as attitudes toward specific aspects of this development. Our findings show that the public is spread out along the attitude spectrum in an evenly manner from strong support to strong opposition on the general question of wind power as energy source. If wind power is proposed located close to residence, close to half the population express a more negative attitude. Recent developments in energy polices in Norway have proven to be premature, or at least progress too quickly to achieve sufficient support, at least among certain groups, or in certain parts of the country. This has led to a temporary moratorium on national-level plans and spurred a revision of the concession process to reach acceptable political compromises and more legitimate energy policies. Based on the findings from this study, it seems clear that both accelerating and reducing the pace of wind power development will continue to create public conflict given that both development paths have large constituencies.

Not surprisingly, the general like- or dislike orientation towards wind power have a strong bearing on the attitudes toward more specific aspects of wind power. For the three main dimensions we included her; place attachment, relations to politics and localization, we find a consistent divergence in attitudes between those who are in favor of and against wind power. Our interpretation is that people who are generally negative to wind power development tend to see this as interventions that have negative practical and emotional impacts to their experience of the landscape. Conversely, supporters of wind power have a positive view of the role of wind power in the total energy mix, the available political instruments, and incentives to promote development, and a stronger faith in the role of wind power in the green transition. The attitude differences between supporters and opposers are also clearly reflected in the attitudes toward localization of windmills. In most aspects, people with a favorable view of wind power have more faith in energy markets and the potential for meeting new energy needs, new developments offshore, and are less concerned about impacts to residential areas.

One of our assumptions was that underlying dimensions of general environmental attitudes would correlate with attitudes toward wind power. Again, we see that people with a dominantly eco-centric orientation place more emphasis on the value of nature and protecting environment, and disagree more that economic needs and energy development should have the right of way. Not surprisingly, supporters of wind power place more weight on the role of technology in solving future energy demands. The latter may be a statement to support wind power to increase the capacity to export energy to other European countries. The findings support our assumption that attitudes toward wind power, at least to some extent, are embedded within broader attitude environmental attitude patterns.

Earlier research on public attitudes toward wind power have in different ways demonstrated the complexity of social acceptance and the importance of varying technical characteristics, as well as social, economic, and environmental impacts. Essentially, the specific context and the way concession and construction processes are performed are critical to the understanding of how attitudes toward wind power are shaped. In this study we also see that the public expresses a range of attitudes from strong support to strong opposition, both on the general level and towards specific aspects of place impacts and the role of politics. We would also argue that the differences and diversity we identify in these patterns toward a specific type of renewable energy development are linked to deeper and different beliefs about what constitutes sustainable energy development and broader environmental attitudes along the anthropocentric – eco-centric continuum.

On the level of national political discourse there is a touching agreement among most political parties. Whether government is dominated by conservatives or liberals makes no difference. No influential politician questions the core idea, embedded in EM, that all environmental challenges should be solved within the framework of economic growth. On the level of popular opinions this is not the case, however. The dominant eco-modernization rhetoric may very well represent one of the challenges for popular community acceptance of wind power projects, as has for instance been observed in the Netherlands (Pohle 2021). Our findings support our assumption that the wind power debate is characterized by a divide, or even antagonism within the broader environmental movement between eco-modernization “optimists” and the de-growth movement. The latter does not recognize the possibility that economic growth can be environmentally sustainable, whilst the first embraces that idea and puts it forward as the pathway to a sustainable future. Attitudes towards windmill developments seem to be fundamentally linked to what might be termed “world views” (Skogen and Krange 2020). We observe a divide within the broad environmental movement: Positive attitudes correspond with technology optimism and the rejection of an antagonistic or unsolvable relationship between growth and nature protection. On the other hand, negative attitudes are associated with more traditional environmental ideas, propagating a slower pace of development, and highlighting the fundamental conflict between economic growth and nature conservation – ultimately expressed as the need for a de-growth movement.

## Conclusion and Policy Implications

Improved knowledge about the public’s attitudes toward wind power development can be vital for future policy, planning and concession processes. Construction of windmill parks can have profound impact on people’s sense of place by changing the way people use and experience their surroundings and the meaning attributed to landscapes. However, different attitude segments of the public experience this in different ways ranging from a highly negative transformation of known places through what is seen as foreign and alienating landscape elements, to positive effects by increasing accessibility through new infrastructure and signs of technological progress and steps toward energy sustainability.

Practical experience during the last few years shows that politicians, land management institutions and developers have underestimated or been outright ignorant of the power of this attitude diversity, to the extent that national authorities were compelled to halt further plans. Currently, this is forcing a policy change in order to award local municipalities a greater say in localization and construction of new windmill plants. With the recent and ongoing conflict in Ukraine which is causing considerable new challenges and demands in the European energy grid, it is almost certain that this will put more pressure on the renewable energy development in Norway. This accentuates the need for policy and concession processes that the public sees as legitimate and transparent. Public trust in responsible institutions and actors driving the wind power arena will be critical to avoid unmanageable conflict. We argue that trust in these types of processes will depend on procedures where the planning and concession processes take into consideration the range of public concerns related to impacts to landscape values and sense of place, perceived fairness in the distribution of costs and benefits of new constructions, the interests of local communities, and that responsible institutions allow sufficient time for proper impact assessments to be conducted.

This study has shown the complexity of wind power attitudes on a national, decontextualized level. We see two implications as particularly important. First, a critical challenge in designing legitimate impact and decision-making processes will be to gather similar type of information in specific sites and contexts influenced by new development plans. Second, wind power development affects a range of societal values linked to large landscapes. Many of these values are non-economic, non-consumptive and non-instrumental values, but nonetheless important and defining to people’s degree of social acceptability. As wind power construction most likely will expand in the future, it becomes imperative to gain a better understanding of how immaterial landscape values are affected, and how this can be fed into ongoing policy processes.
