# Correction: Attitudes toward Wind Power in Norway–Solution or Problem in Renewable Energy Development?

**DOI:** 10.1007/s00267-024-01986-2

**Published:** 2024-05-16

**Authors:** Bjørn P. Kaltenborn, Rose Keller, Olve Krange

**Affiliations:** 1 Norwegian Institute for Nature Research, Vormstuguvegen 40, 2624 Lillehammer, Norway; 2 Norwegian Institute for Nature Research, Sognsveien 58, 0855 Oslo, Norway

Correction to: Environmental Management (2023) 72:922–931

10.1007/s00267-023-01870-5

In this article under the section heading ‘’Wind Power Development in Norway’’ in the first paragraph the sentence ‘’Currently 70 wind power plants with 13054 turbines are spread out along large sections of the Norwegian coastland and some inland locations with visual, audial, and cumulative environmental impacts far beyond the construction zones (NVE 2022).’’ has been corrected to ‘’Currently 70 wind power plants with 1354 turbines are spread out along large sections of the Norwegian coastland and some inland locations with visual, audial, and cumulative environmental impacts far beyond the construction zones (NVE 2022).’’

The original article has been corrected.

